# Prediction of overall survival based upon a new ferroptosis-related gene signature in patients with clear cell renal cell carcinoma

**DOI:** 10.1186/s12957-022-02555-9

**Published:** 2022-04-14

**Authors:** Zhuolun Sun, Tengcheng Li, Chutian Xiao, Shaozhong Zou, Mingxiao Zhang, Qiwei Zhang, Zhenqing Wang, Hailun Zhan, Hua Wang

**Affiliations:** 1 Department of Urology, Third Affiliated Hospital of Sun Yat-sen University, Guangzhou, 510630 China; 2 College of Life Science and Technology, Jinan University, Guangzhou, 510630 China; 3 Department of Urology, The First Affiliated Hospital of Sun Yat-sen University, Guangzhou, 510630 China; 4 Department of Thoracic Surgery, The Seventh Affiliated Hospital, Sun Yat-sen University, Shenzhen, 518107 China

**Keywords:** Clear cell renal cell carcinoma, Ferroptosis, Prognostic signature, Nomogram, Bioinformatics

## Abstract

**Background:**

Clear cell renal cell carcinoma (ccRCC) is the most common and lethal renal cell carcinoma (RCC) histological subtype. Ferroptosis is a newly discovered programmed cell death and serves an essential role in tumor occurrence and development. The purpose of this study is to analyze ferroptosis-related gene (FRG) expression profiles and to construct a multi-gene signature for predicting the prognosis of ccRCC patients.

**Methods:**

RNA-sequencing data and clinicopathological data of ccRCC patients were downloaded from The Cancer Genome Atlas (TCGA). Differentially expressed FRGs between ccRCC and normal tissues were identified using ‘limma’ package in R. GO and KEGG enrichment analyses were conducted to elucidate the biological functions and pathways of differentially expressed FRGs. Consensus clustering was used to investigate the relationship between the expression of FRGs and clinical phenotypes. Univariate and the least absolute shrinkage and selection operator (LASSO) Cox regression analysis were used to screen genes related to prognosis and construct the optimal signature. Then, a nomogram was established to predict individual survival probability by combining clinical features and prognostic signature.

**Results:**

A total of 19 differentially expressed FRGs were identified. Consensus clustering identified two clusters of ccRCC patients with distinguished prognostic. Functional analysis revealed that metabolism-related pathways were enriched, especially lipid metabolism. A 7-gene ferroptosis-related prognostic signature was constructed to stratify the TCGA training cohort into high- and low-risk groups where the prognosis was significantly worse in the high-risk group. The signature was identified as an independent prognostic indicator for ccRCC. These findings were validated in the testing cohort, the entire cohort, and the International Cancer Genome Consortium (ICGC) cohort. We further demonstrated that the signature-based risk score was highly associated with the ccRCC progression. Further stratified survival analysis showed that the high-risk group had a significantly lower overall survival (OS) rate than those in the low-risk group. Moreover, we constructed a nomogram that had a strong ability to forecast the OS of the ccRCC patients.

**Conclusions:**

We constructed a ferroptosis-related prognostic signature, which might provide a reliable prognosis assessment tool for the clinician to guide clinical decision-making and outcomes research.

**Supplementary Information:**

The online version contains supplementary material available at 10.1186/s12957-022-02555-9.

## Introduction

Renal cell carcinoma (RCC) is one of the most common types of urological tumors, accounting for almost 3% of all adult malignancies in western countries [1]. The incidence of RCC is increasing at an annual rate of 3–5%, with an estimated 140,000 kidney cancer-related deaths per year [2, 3]. Clear cell renal cell carcinoma (ccRCC) is the most common and lethal subtype of RCC and accounts for 90% of all kidney cancers [1]. Despite improvements in the surgery and other comprehensive treatment methods, the clinical outcomes for ccRCC remain unsatisfactory, with a median overall survival (OS) ranging from 16 to 50 months based on site of metastatic involvement [4, 5]. Surgical resection remains the definitive treatment for patients with localized disease. Approximately 30% of patients present metastatic at the time of diagnosis, which requires systemic therapies and is associated with high mortality [2]. The complexity and heterogeneity of ccRCC have made prognostication and choice of treatment strategy difficult [6]. In ccRCC patients, the tumor grade at the time of diagnosis may affect the survival rate. The 5-year tumor-specific mortality rate for grade 1 patients is about 7%, and that for grade 4 patients is about 58% [7]. Therefore, to improve the therapeutic outcomes and life quality of patients, there is an additional need for developing more effective biomarkers for early screening and diagnosis.

Programmed cell death (PCD) is a fundamental self-destruction process in cell development and growth, which is widely considered a positive process that both prevents and treats cancer [8]. However, abundant studies have demonstrated that PCD can also cause unwanted effects that may even promote tumorigenesis, progression, and metastasis [9–11]. Ferroptosis is a recently discovered type of PCD, which is characterized by the lipid peroxidation caused by iron accumulation [12]. Ferroptosis is closely related to the metabolism of iron, fatty acids, amino acids, as well as the biosynthesis of glutathione, phospholipids, and NADPH [13, 14]. The iron metabolism and lipid peroxidation are reported to be two pivotal mechanisms of ferroptosis [15]. Preliminary evidence suggests that ferroptosis may have a tumor suppressor function that could be potentially beneficial for cancer therapy [13]. On the other hand, substantial studies have also shown the crucial role of ferroptosis in tumor initiation and progression [16–19]. For example, some authors evaluated the *GPX4* expression in HCC tissue samples and verified that *GPX4* was significantly over-expression and associated with an increased malignancy grade [20]. There is longstanding evidence that various primary tumors and also metastases express DPP4 to a variable extent [21]. In addition, other ferroptosis regulatory genes such as S1R [22], NRF2 [23], and NFS1 [24] have also been shown to be strongly correlated with tumorigenesis and progression. Nevertheless, whether the ferroptosis-related genes (FRGs) affect the prognosis of ccRCC patients has not been investigated in detail.

In the present study, we performed a genome-wide comparative analysis of FRGs expression profiles and investigated differentiated FRGs expression patterns in ccRCC patients based on The Cancer Genome Atlas (TCGA) and The International Cancer Genome Consortium (ICGC) databases. We constructed a 7-gene signature that could predict the outcome of ccRCC patients. Our results demonstrate that some FRGs play vital roles in ccRCC progression, which might serve as potential prognostic biomarkers and therapeutic targets for ccRCC patients.

We present the following article in accordance with the MDAR reporting checklist.

## Materials and methods

### Data acquisition

We obtained the RNA-sequencing data of 72 normal kidney and 539 KIPC samples with corresponding clinical information from TCGA (https://cancergenome.nih.gov/) database. We excluded cases (*n* = 5) without follow-up records (survival time code of 0 months). Time to follow-up ranges from 0.07 to 122.27 months with an average length of 37.90 months in this study. Patients (*n* = 48) with incomplete clinical data were excluded from this analysis. RNA-seq data and clinical information of another 90 ccRCC were downloaded from ICGC (https://dcc.icgc.org/) database, which was used as an independent external validation set.

We searched the previous literature to identify 61 ferroptosis-related genes (FRGs) described so far to be involved in ferroptosis [13, 18, 25]. The list of genes is presented in Table S1. The expression data of the FRGs were extracted and used for subsequent analysis.

### Data preprocessing and differentially expressed FRGs screening in ccRCC

Raw expression data were background corrected, quantile normalized, and logarithmic conversion using the R language. The ensemble gene IDs were then converted to gene symbols through the GRCh38 reference genome (http://asia.ensembl.org/index.html) in this study. The tumor and normal tissues were compared to identify differentially expressed FRGs based on the significance threshold of |logFC (fold change)| > 1 and a false discovery rate (FDR) < 0.05. Next, these differentially expressed FRGs were subjected to construct protein-protein interaction (PPI) network on the Search Tool for the Retrieval of Interacting Genes/Proteins (STRING, http://string.embl.de/) [26]. Visualization was then rendered using Cytoscape (http://www.cytoscape.org/).

### Functional enrichment analysis

The biological processes (BP), cellular components (CC), and molecular functions (MF) of the differently expressed FRGs were examined using gene ontology (GO) enrichment analysis. The Kyoto Encyclopedia of Genes and Genomes (KEGG) pathway enrichment analysis was applied to explore the significant pathways of differentially expressed FRGs. All enrichment analyses were conducted with the ‘ClusterProfiler’ R package [27]. A *P* value < 0.05 was regarded as statistically significant.

GSCALite (http://bioinfo.life.hust.edu.cn/web/GSCALite/) database represents an important online platform, which can help the cancer research community to discover cancer pathways and drugs [28]. In the current research, we also used the GSCALite database to determine the degree of gene activation or inhibition of classical pathways.

### Non-negative matrix factorization consensus clustering

To study the association between the expression profiles of FRGs and clinical subtypes in ccRCC, we clustered the ccRCC cohort into diverse clusters by consensus clustering with ‘ConsensusClusterPlus’ in R [29]. Principal component analysis (PCA) was carried out to evaluate the gene expression patterns in the different clusters. We then compared the OS difference between clusters by the Kaplan–Meier survival analysis in R. Chi-square test was used to compare the frequency distribution of age, gender, grade, AJCC stage, and TNM stage between different clusters. No analysis was performed on the N stage owing to some missing data.

### Construction and validation of the ferroptosis-related prognostic risk signature

The expression data of the differentially expressed FRGs were explored using the univariate Cox regression analysis to screen FRGs with prognostic values (*P* < 0.05). The TCGA-ccRCC dataset was then randomly divided into a training cohort (*n* = 294) and a testing cohort (*n* = 192) for subsequent validation. The least absolute shrinkage and selection operator (LASSO) Cox regression was then conducted to construct a prognostic signature within the training cohort. We repeated the simulations 1000 times for which the optimal penalty parameter (λ) was identified via 10-fold cross validation following the minimum criteria. In addition, only genes with non-zero coefficients were chosen to further calculate the risk score. The risk score was estimated using the following formula: Risk Score$={\sum}_{i=1}^n\left(\mathrm{Exp}i\ast \mathrm{Coe}i\right)$. N, Expi, and Coei represented gene number, level of gene expression, and coefficient value, respectively. The median risk score was chosen as a cutoff value to dichotomize the training cohort into high- and low-risk groups. The Kaplan–Meier survival curve was used to evaluate the differences in OS between the two groups by the log-rank test. Additionally, the receiver operating characteristic (ROC) curve and the area under the ROC curve (AUC value) was applied to estimate the accuracy of the prognostic signature [30]. To determine whether risk score was an independent prognostic factor, the univariate and multivariate Cox regression analyses were performed. The Kaplan–Meier survival curve of the individual gene in the signature was analyzed using the optimum cut-off value through the X-Tile software [31]. Besides, the testing cohort, the entire cohort, and the ICGC cohort were applied as validation cohorts to verify the predictive capacity of the constructed signature according to the same formula.

### The clinical application of the ferroptosis-related prognostic signature

To test the usability and feasibility of the signature in the clinic, the relationship between the ferroptosis-related risk signature and clinical parameters in TCGA-ccRCC patients was performed. We explored the power of the ferroptosis-related prognostic signature to predict the survival stratified by various clinical characteristics using the Kaplan–Meier analysis. In addition to this, a nomogram integrated the prognostic signature and clinical parameters, was constructed as a quantitative prediction tool to evaluate clinical prognosis [32]. Following that, calibration curves were generated to evaluate the concordance between actual and predicted survival. Moreover, the concordance index (C-index) was computed to evaluate the model performance for predicting prognosis, with a C-index of 1 indicating perfect discrimination and a C-index of 0.5 indicating a random guess. Decision curve analysis (DCA) was applied to assess the clinical usefulness of the nomogram by calculating the net benefits for a range of threshold probabilities.

### The expression patterns, SNVs, CNVs, and drug sensitivity of the genes in the signature

To confirm the reliability of the genes in the signature, we verified the expression in different pathological tumors based on the data from the TCGA database. GSCALite database was then employed to explore single nucleotide variations (SNVs) and copy number variations (CNVs) of the genes in the signature in ccRCC patients.

CellMiner (https://discover.nci.nih.gov/cellminer/home.do) database was used to examine the expression levels of the signature-related genes in the different ccRCC cells and the resulting values were represented as a heatmap. To provide support for the drug selection of gene targeting therapy, we then analyzed the correlation of gene expression and drug sensitivity in ccRCC patients with the GSCALite database.

## Results

### Differentially expressed FRGs in ccRCC

**Fig. 1:**
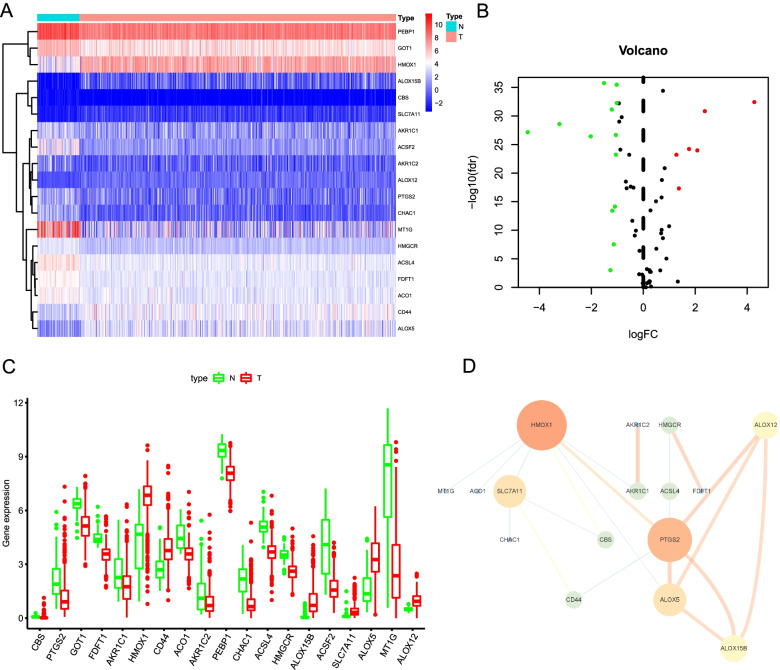
Identification of differentially expressed FRGs in the TCGA database. **A** The heatmap demonstrates the expression of 19 differentially expressed FRGs in ccRCC samples compared to normal samples. **B** Volcano plot of 19 differentially expressed FRGs in ccRCC samples compared to normal samples. **C** The box diagram exhibits the expression patterns, median values, and data ranges of the 19 differentially expressed FRGs. **D** The correlation network of the 19 differentially expressed FRGs

We first investigated the expression levels of 61 FRGs in ccRCC and normal samples based on the TCGA database. A total of 19 differentially expressed FRGs was eventually determined, including 13 downregulated genes (MT1G, ACSF2, CHAC1, ACSL4, AKR1C2, PEBP1, PTGS2, AKR1C1, CBS, GOT1, ACO1, FDFT1, and HMGCR) and 6 upregulated genes (ALOX12, CD44, SLC7A11, ALOX5, HMOX1, and ALOX15B) in ccRCC tissues (Fig. 1A and B). The box diagram was utilized to exhibit the expression patterns, median values, and data ranges of the differentially expressed FRGs in tumor and non-tumor cases (Fig. 1C). The interaction network among these genes was presented in Fig. 1D, and the result indicated that PTGS2 and HMOX1 seemed to be the hub genes in this network.

### Two ferroptosis subgroups were different in clinical phenotypes and OS via consensus clustering analysis

**Fig. 2:**
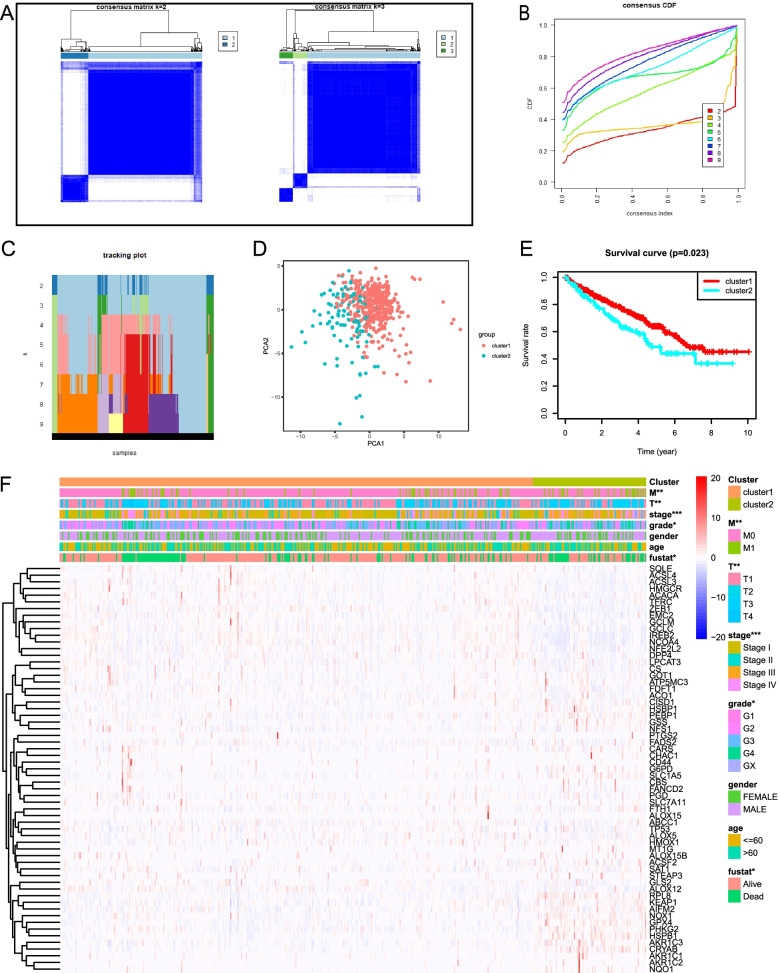
Consensus clustering analysis of FRGs expression profiles of the ccRCC patients in the TCGA dataset. **A** The consensus maps show the consensus clustering matrix for FRGs in the ccRCC dataset for *k* = 2, 3. The optimal clustering is represented by *k* = 2. **B** Consensus clustering cumulative distribution function (CDF) for *k* = 2 to 9. **C** The tracking plot for *k* = 2 to 9. **D** Principal component analysis demonstrates the gene expression differences between two clusters (cluster1 and cluster 2). **E** The OS in cluster 2 is significantly shorter than that in cluster 1. **F** Heatmap shows the clinical features between the two ccRCC patient clusters. *P < 0.05; **P < 0.01; ***P < 0.001

According to expression levels of 61 FRGs, the ccRCC patients were clustered into 2 subtypes (cluster 1 and cluster 2) with *k* = 2 as the optimal value as the grouping was suboptimal when they were classified into more than 2 clusters (Fig. 2A–C). Moreover, PCA was performed to compare the transcriptional profile between cluster 1 and cluster 2. The result demonstrated that there was a significant distinction between the two subgroups (Fig. 2D). We compared the OS between two clusters and observed that cluster 2 had a shorter OS than cluster 1 for the ccRCC patients (Fig. 2E). We then evaluated associations between the clustering and the clinicopathological parameters of ccRCC patients. The result showed that there were differences in grade (*P* < 0.05), AJCC stage (*P* < 0.001), T stage (*P* < 0.01), M stage (*P* < 0.01), and survival status (*P* < 0.05) between two clusters, but did not present any differ significantly in age and gender (Fig. 2F). Therefore, these results suggested that ferroptosis was closely related to clinical phenotypes and the progression of ccRCC.

### Functional enrichment analysis of the differentially expressed FRGs

**Fig. 3:**
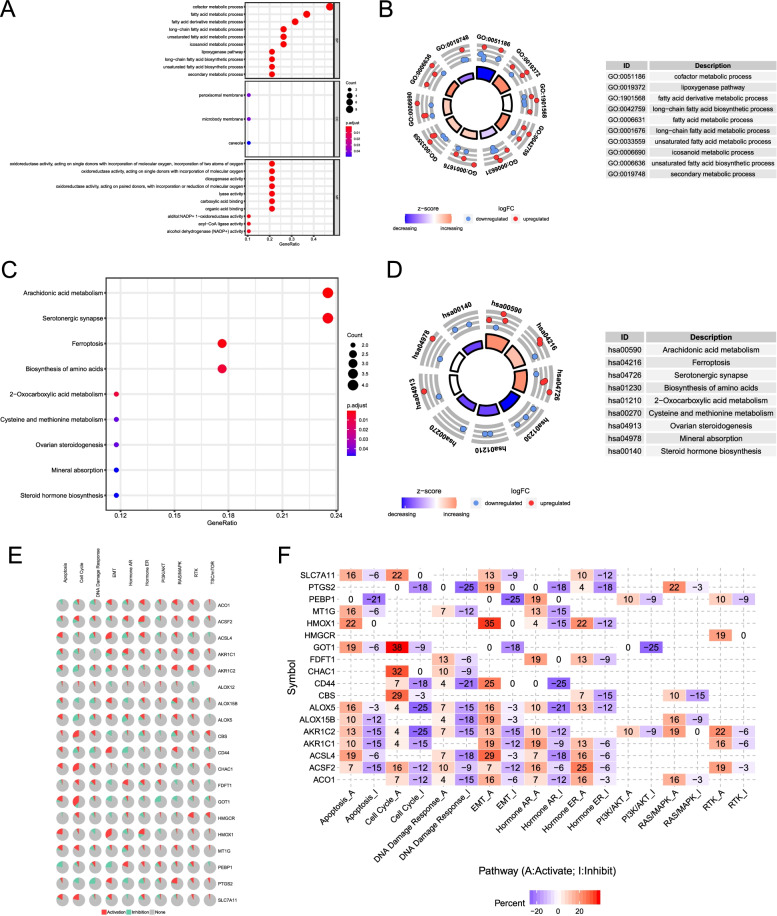
GO and KEGG functional annotation analyses of 19 differentially expressed ferroptosis-related genes. **A** Bubble chart of significantly enriched GO term. **B** The circle shows the scatter map of the specified gene in each GO term. The red circles and blue circles represent upregulation and downregulation genes, respectively. The higher the Z-score value, the higher the enrichment pathway expression. **C** Bubble chart of significantly enriched KEGG pathways. **D** The circle shows the scatter map of the specified gene in each KEGG pathway. The red circles and blue circles represent upregulation and downregulation genes, respectively. The higher the Z-score value, the higher the enrichment pathway expression. **E** The pie chart of the correlation between differentially expressed FRGs and classical cancer pathways. Red color represents activates pathways and green color represents inhibits pathways. **F** Network diagram of the correlation between differentially expressed FRGs and classical cancer pathways. Red color represents activates pathways and blue color represents inhibits pathways

To elucidate the biological functions and pathways of the 19 differentially expressed FRGs, GO functional annotation and KEGG pathway enrichment analyses were conducted. The results revealed that these differently expressed FRGs were significantly enriched in the BP related to several metabolic processes, such as cofactor metabolic process, fatty acid metabolic process, and fatty acid derivative metabolic process (Fig. 3A and B). Furthermore, the lipoxygenase pathway was also involved. In terms of CC, we found that the differently expressed FRGs were significantly enriched in peroxisomal membrane, microbody membrane, and caveola. Through the MF, the differently expressed FRGs were notably related to oxidoreductase activity, dioxygenase activity, and lyase activity. In the KEGG pathway enrichment analysis, these genes were shown to be mostly associated with pathways in arachidonic acid metabolism, serotonergic synapse, and ferroptosis (Fig. 3C and D). In addition, we explored the effect of the differentially expressed FRGs in multiple classical signaling pathways on ccRCC using the GSLA database. The results revealed that some FRGs were associated with the activation or inhibition of oncogenic pathways (Fig. 3E and F).

### Prognosis-related FRGs selecting and construction of a prognostic signature based on seven FRGs in the training cohort

**Fig. 4:**
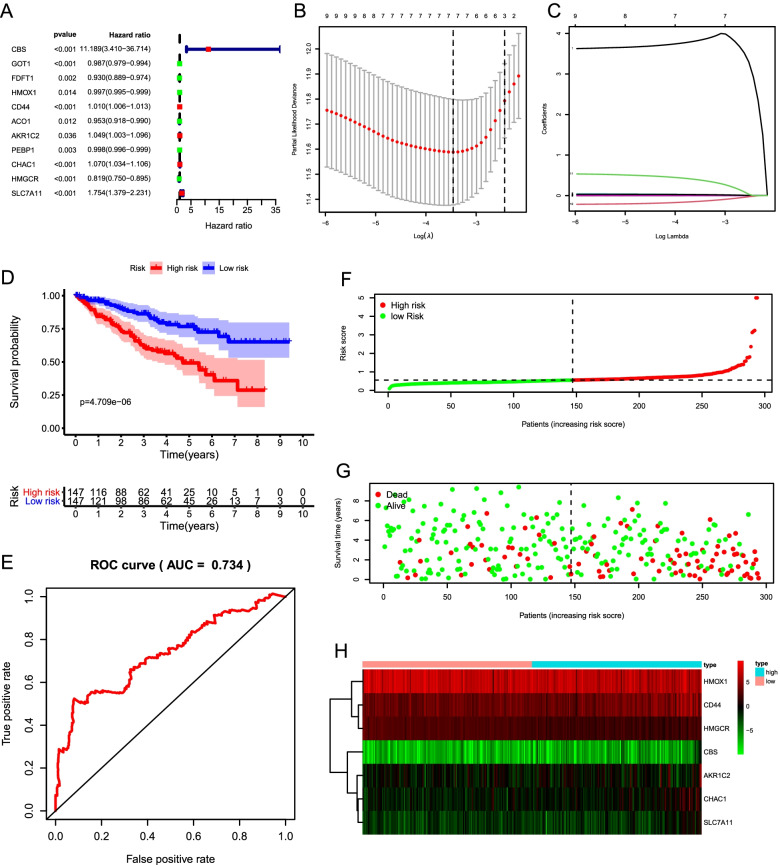
Construction and evaluation of a prognostic signature based on seven FRGs in the training cohort. **A** Univariate Cox analysis reveals 11 candidate prognosis-related FRGs. **B** The optimal penalty parameter (λ) at the vertical line is selected following the minimum criteria (left). **C** Lasso coefficient profiles of the 11 candidates prognosis-related FRGs. **D** Kaplan–Meier curve shows that the high-risk group had significantly shorter OS than the low-risk group. **E** ROC curve demonstrates the veracity and reliability of the signature. **F** Risk score distribution of TCGA-ccRCC patients. **G** The distributions of risk scores and the survival status. **H** The heatmap displays that the expression levels of the seven FRGs in the high- and low-risk groups

We conducted a univariate Cox regression analysis and found that expression levels of 11 FRGs (CBS, GOT1, FDFT1, HMOX1, CD44, ACO1, AKR1C2, PEBP1, CHAC1, HMGCR, and SLC7A11) were closely correlated with the OS (*P* < 0.05; Fig. 4A). Genes (CBS, CD44, AKR1C2, CHAC1, and SLC7A11) with HR > 1 were considered as risk genes, while the remaining six genes (GOT1, FDFT1, HMOX1, ACO1, PEBP1, and HMGCR) with HR < 1 as protective genes. According to the expression of the eleven genes mentioned above in the training cohort, a prognostic risk signature was constructed using LASSO Cox regression analysis. As a result, a 7-gene prognostic signature (CBS, HMOX1, CD44, AKR1C2, CHAC1, HMGCR, and SLC7A11) was identified (Fig. 4B and C). Survival analyses based on the optimal cut-off expression of the individual gene showed that high expression of risk genes (CBS, CD44, AKR1C2, CHAC1, and SLC7A11) was correlated with poor prognosis, while high expression of protective genes (HMOX1 and HMGCR) displayed the opposite patterns (Fig. S1).

Based on the 7 candidate FRGs, the risk score of each patient was calculated according to the following formula: risk score = (3.8463 × CBS) + (− 0.0021 × HMOX1) + (0.0101 × CD44) + (0.0208 × AKR1C2) + (0.0252 × CHAC1) + (− 0.1354 × HMGCR) + (0.3774 × SLC7A11). We then used the median risk score as a cutoff point for classifying ccRCC patients in the training cohort (*n* = 294) into high-risk group (*n* = 147) and low-risk group (*n* = 147). Kaplan–Meier survival curve analysis showed that OS was significantly different between the predicted two risk groups and the high-risk group had a significantly shorter survival time compared to the low-risk group (*P* = 4.709e-06; Fig. 4D). The ROC curve analysis suggested the risk signature had a promising predictive value for ccRCC survival prediction (AUC = 0.734) (Fig. 4E). We then ranked the risk scores of the patients and then analyzed their distributions (Fig. 4F). Compared to the high-risk group, the distributions of risk scores and the survival status showed survival rate and time were significantly increased in the low-risk group (Fig. 4G). The heatmap displayed the expression of the seven candidate FRGs between two groups (Fig. 4H).

### Validation of the ferroptosis-related signature in the testing cohort, the entire cohort, and the ICGC cohort

**Fig. 5:**
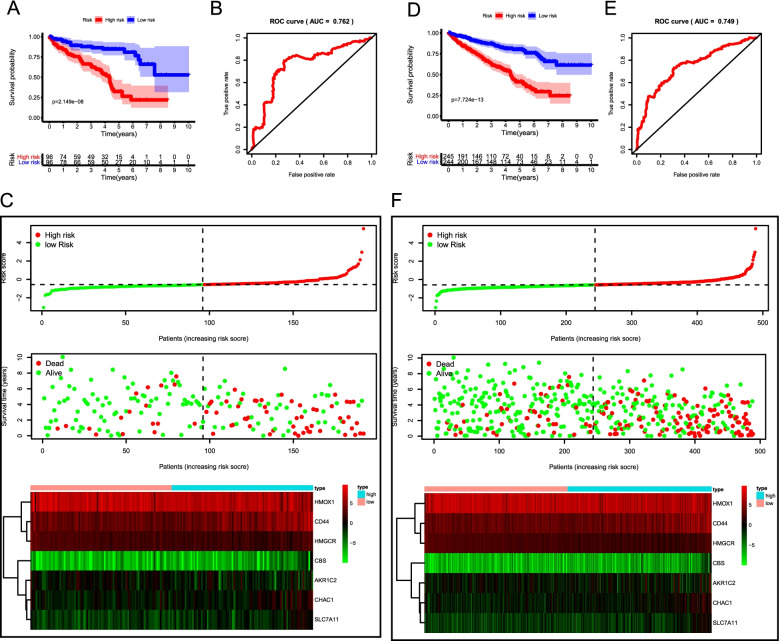
Validation of the prognostic risk signature in the testing cohort and entire cohort. Kaplan–Meier curve analysis of high-risk and low-risk patients in the testing cohort (**A**) and the entire TCGA cohort (**D**). ROC curve analysis of the testing cohort (**B**) and the entire TCGA cohort (**E**). The risk score distribution, survival status, and risk gene expression in the testing cohort (**C**) and the entire TCGA cohort (**F**)

To verify the accuracy and robustness of the prognostic risk signature, the predictive ability of this signature was further validated in the testing cohort (*n* = 192), the entire cohort (*n* = 486). The prognostic risk score was calculated for patients in each cohort according to the prognostic signature. The detailed clinical features of ccRCC patients were listed in Table 1. We observed that the results in the testing and entire cohort were consistent with the outcome in the training cohort. Kaplan–Meier survival curve revealed that patients in the high-risk group presented worse OS than their low-risk counterparts in both the testing cohort (*P* = 2.149e-08) (Fig. 5A) and the entire TCGA-ccRCC cohort (*P* = 7.724e-13) (Fig. 5D). ROC curve analysis indicated that the AUC values in the testing cohort and the entire TCGA- ccRCC cohort were 0.762 (Fig. 5B) and 0.749 (Fig. 5E), respectively. The distribution of risk score, survival result, and the seven gene expression heatmap in the testing cohort and entire cohort were shown in Fig. 5C and Fig. 5F.

**Table 1:** Characteristics of ccRCC patients included in this study

Variable	Training cohort (*n* = 294)	Testing cohort (*n* = 192)	TCGA cohort (*n* = 486)	***P***
	Number (%)	Number (%)	Number (%)	
**Age**				0.9261
≤ 60	148 (50.34)	95 (49.48)	243 (50.00)	
> 60	146 (49.66)	97 (50.52)	243 (50.00)	
**Gender**				0.6231
Female	102 (34.69)	62 (32.29)	164 (33.74)	
Male	192 (65.31)	130 (67.71)	322 (66.26)	
**Grade**				0.1454
G1	7 (2.38)	3 (1.56)	10 (2.06)	
G2	125 (42.52)	85 (44.27)	210 (43.21)	
G3	126 (42.86)	68 (35.42)	194 (39.92)	
G4	36 (12.24)	36 (18.75)	72 (14.81)	
**AJCC stage**				0.9623
I	142 (48.3)	94 (48.96)	236 (48.56)	
II	32 (10.88)	18 (9.38)	50 (10.29)	
III	72 (24.49)	48 (25)	120 (24.69)	
IV	48 (16.33)	32 (16.67)	80 (16.46)	
**T stage**				0.5685
T1	144 (48.98)	98 (51.04)	242 (49.79)	
T2	41 (13.95)	20 (10.42)	61 (12.55)	
T3	101 (34.35)	71 (36.98)	172 (35.39)	
T4	8 (2.72)	3 (1.56)	11 (2.26)	
**M stage**				0.9837
M0	248 (84.35%)	161 (83.85%)	409 (84.16%)	
M1	46 (15.65%)	31 (16.15%)	77 (15.84%)

Then the prognostic signature was validated in the ICGC cohort (*n* = 90). The OS was significantly poorer in the high-risk group than in the low-risk group (*P* = 1.592e-02) (Fig. S2A). The AUC value for the prognostic signature was 0.71, suggesting well-prediction performances (Fig. S2B). The distribution of risk score, survival status, and gene expression of ccRCC patients in the ICGC cohort was presented in Fig. S2C, which were similar to the above cohorts. Taken together, these results revealed that the risk signature could accurately predict the clinical outcomes of ccRCC patients.

### The ferroptosis-related signature was an independent prognostic indicator

To identify whether the ferroptosis-related signature could serve as an independent prognostic indicator, univariate and multivariate Cox regression analyses were conducted with the risk score and clinical parameters. In the TCGA training cohort, univariate analyses showed that the age and risk score were significantly related to OS. Besides, subsequent multivariate analyses suggested that the age and risk score were still strongly related to OS (Table 2). Both the testing and the entire cohorts yielded very similar results (Table 2). Therefore, the signature-based risk score was an independent adverse prognostic indicator for OS in ccRCC patients.

**Table 2:** Univariate and multivariate Cox regression analysis of clinical factors and prognostic risk signature in the training cohort, the testing cohort and the entire cohort

Variable	Training cohort				Testing cohort				Entire cohort			
	Univariate		Multivariate		Univariate		Multivariate		Univariate		Multivariate	
	HR	*P*	HR	*P*	HR	*P*	HR	*P*	HR	*P*	HR	*P*
**Age**: ≤ 65 vs. > 65	1.03	1.23e-03	1.04	1.10e-04	1.03	5.91e-03	1.04	9.28e-04	1.03	1.24e-05	1.04	2.98e-07
**Gender**: Female vs. Male	0.95	0.82	1.20	0.43	0.91	0.70	0.78	0.36	0.94	0.70	1.03	0.87
**Grade**: G1–2 vs. G3–4	2.05	7.1e-07	1.27	0.16	2.72	3.51e-09	1.67	7.49e-03	2.30	2.24e-14	1.31	3.09e-02
**AJCC stage**: I/II vs. III/IV	1.95	3.4e-13	1.75	0.08	1.91	3.42e-09	2.17	8.13e-03	1.93	9.60e-21	1.98	1.40e-03
**T stage**: T1–2 vs. T3–4	2.14	2.92e-11	1.00	0.99	1.80	1.35e-05	0.58	4.80e-02	1.99	3.80e-15	0.73	0.11
**M stage**: M0 vs. M1	4.07	1.76e-10	0.96	0.94	5.05	8.65e-11	1.38	0.50	4.39	2.68e-19	1.13	0.75
**Risk score**: Low vs. High	1.42	6.65e-12	1.46	5.13e-11	1.78	6.33e-08	1.68	4.14e-04	2.08	8.98e-22	2.04	6.91e-14

### Prognostic risk score indicated strong associations with clinical characteristics in ccRCC

**Fig. 6:**
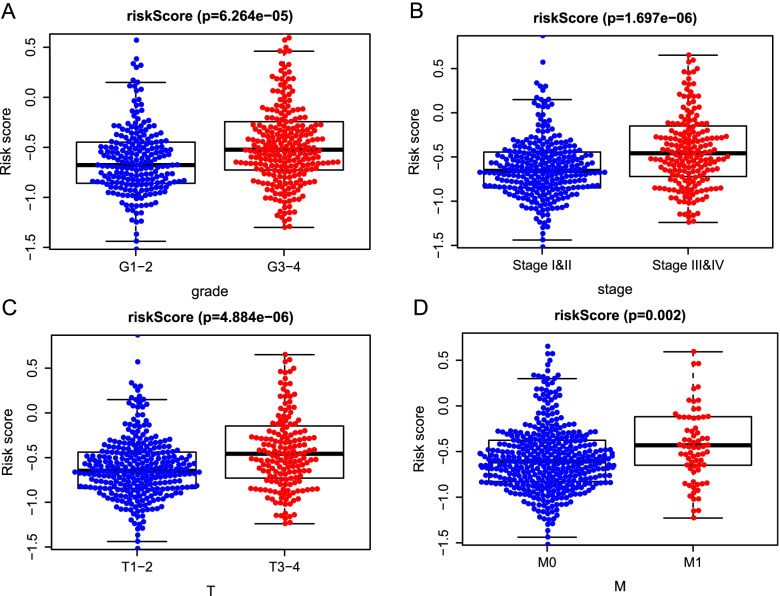
The relationship between the risk score and clinicopathological parameters. The distribution of risk scores between high- and low-risk patients was stratified according to **A** Grade, **B** AJCC stage, **C** T stage, and **D** M stage

To explore whether the prognostic signature could better predict KIPC clinicopathological characteristics, an analysis was performed to explore the associations between the risk signature and clinical parameters. Significant differences were observed between two groups in grade (*P* = 6.264e-06) (Fig. 6A), AJCC stage (*P* = 1.6973–06) (Fig. 6B), T stage (*P* = 4.884e-06) (Fig. 6C), and M stage (*P* = 0.002) (Fig. 6D). Simultaneously, we observed that the advanced-stage tumor was closely linked to the high-risk patients; however, the early-stage tumors were closely linked to the low-risk patients.

**Fig. 7:**
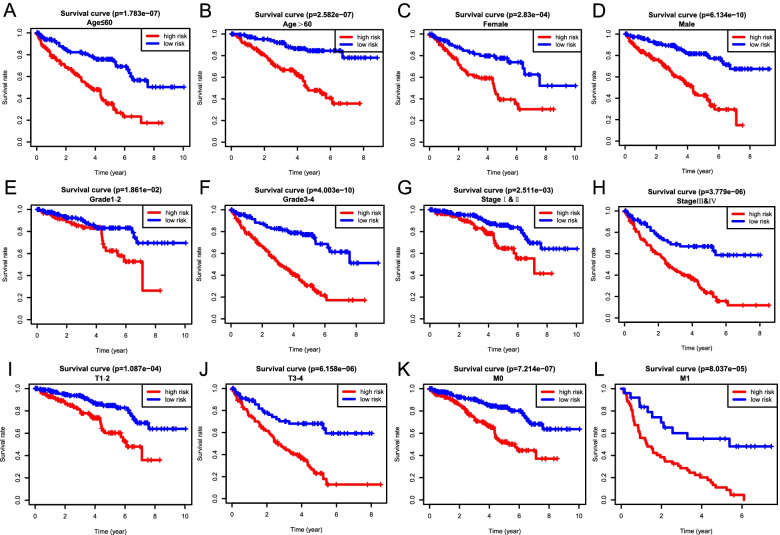
Kaplan–Meier survival analysis of the OS in high- and low-risk ccRCC patients stratified by clinical parameters. **A**, **B** Age (≤ 60 and > 60), **C**, **D** gender (female and male), **E**, **F** grade (G1–2 and G3–4), **G**, **H** AJCC stages (stages I/II and III/IV), **I**, **J** T stages (T1–2 and T3–4), and **K**, **L** M stages (M0 and M1)

To investigate the prognostic value of our ferroptosis-related signature in different subgroups, we conducted stratified survival analysis with the following clinical parameters: age (≤ 60 and > 60), gender (female and male), tumor grade (G1–2 and G3–4), AJCC stage (I & II and III & IV), T stage (T1–2 and T3–4), and M stage (M0 and M1). Interestingly, survival analysis indicated the high-risk group suffered an obviously lower OS than those in the low-risk group for all hierarchical cohorts (Fig. 7). Thus, these findings suggested the classification of the risk signature might be applied to precisely determine the patients with poor prognosis, regardless the clinical parameters.

### Development of a personalized prognostic nomogram

**Fig. 8:**
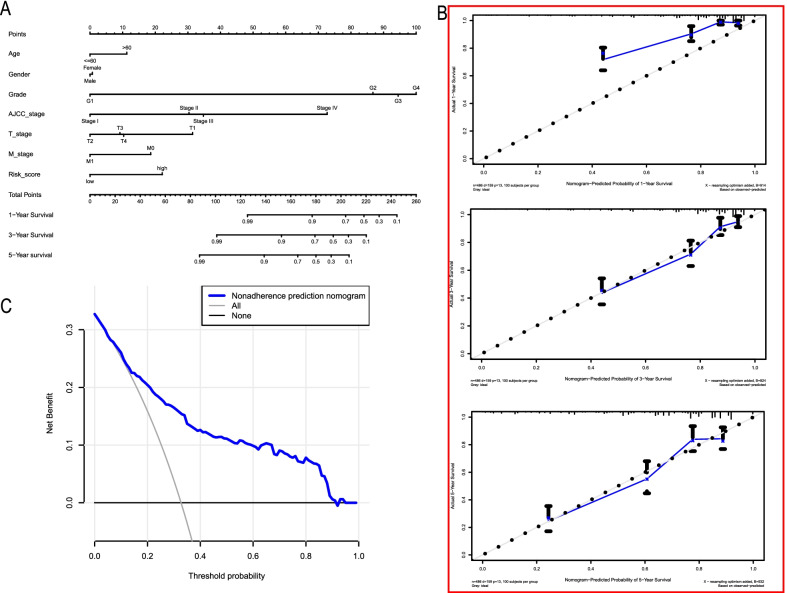
Development and validation of the nomogram predicting OS for ccRCC patients in the TCGA cohort. **A** Prognostic nomogram for ccRCC patients based on the constructed prognostic signature and clinicopathological parameters. **B** Calibration curves of the nomogram to predict OS at 1, 3, and 5 years. **C** The DCA curve of the nomogram

A nomogram is a powerful tool that has been extensively applied to quantitatively determine individuals’ risk in clinical decision-making by incorporating multiple clinical factors [33]. To establish a viable method for predicting survival in ccRCC patients, we developed a prognostic nomogram based on the constructed prognostic risk signature and several clinical features. The nomogram was devoted to estimating the probability of 1-, 3-, and 5-year survival (Fig. 8A). Each factor was assigned a score in proportion to its risk contribution to survival. The C-index used to evaluate the OS of the nomogram was 0.771. Calibration curves showed optimal agreement when compared with an ideal model (Fig. 8B), particularly for 3- and 5-year survival predicted probabilities. DCA indicated that the nomogram had a wide and practical range of threshold probability for the TCGA-ccRCC cohort for predicting survival rates (Fig. 8C).

### The expression patterns, SNVs, CNVs of the seven candidate genes in the signature for ccRCC patients

**Fig. 9:**
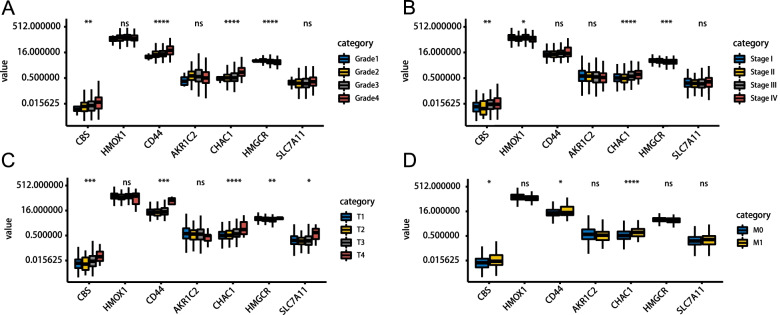
The expression patterns of the seven prognostic FRGs. Expression of CBS, HMOX1, CD44, AKR1C2, CHAC1, HMGCR, and SLC7A11 in ccRCC samples with different Grade (**A**), AJCC stage (**B**), T stage (**C**), and M grade (**D**)

Additionally, we explored the association between the expression level of individual signature-related genes and clinicopathological parameters. In terms of grade alone, CBS, CD44, and CHAC1 increased with tumor grade, while HMGCR was decreased. No significant difference in the expression of HMOX1, AKR1C2, and SLC7A11 was detected between different tumor grades (Fig. 9A). As for different AJCC stage, CBS, HMGCR, CHAC1, and HMOX1 were significantly differentially expressed, with higher expression levels of CBS and CHAC1 indicating higher advanced AJCC stage, while HMGCR and HMOX1 showed the opposite trend (Fig. 9B). Regarding the T stage, it was noted that CBS, CD44, CHAC1, and SLC7A1 were significantly up-regulated in advanced T grade, whereas HMGCR was significantly down-regulated (Fig. 9C). CBS, CD44, and HMGCR also represented similar trends in the N stage as the T stage (Fig. 9D). Taken together, the expression of CBS, CD44, CHAC1, and SLC7A1 were positively associated with tumor progression, while HMOX1 and HMGCR were negatively associated with tumor progression, which was in line with the above study. In addition, AKR1C2 expression appeared to be independent of tumor progression.

We then used the GSCALite database to study the SNVs of the seven candidate genes in the signature for ccRCC patients. The results indicated that the most frequent mutation type was single nucleotide polymorphism (SNP) (Fig. S3A), and missense mutation was the most fraction among these mutations (Fig. S3B). In addition, C > T transversion was the most common type of SNV (Fig. S3C). The characteristic of the frequently mutated genes was showed in Fig. S3D. We also analyzed the CNVs of the seven candidate genes in the signature and found heterozygous mutations (amplification and deletion) in all genes (Fig. S3E).

### Drug sensitivity of the seven candidate genes in the signature for ccRCC patients

**Fig. 10:**
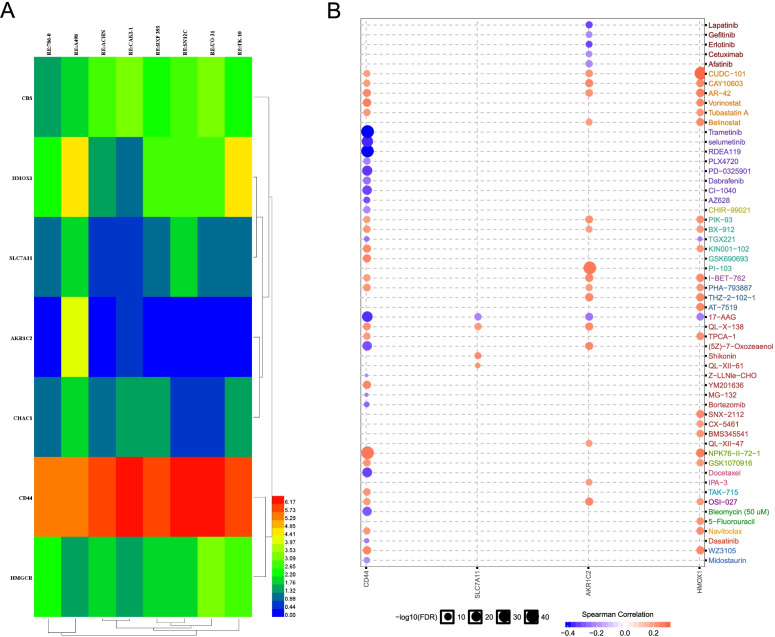
Association of the seven candidate FRGs expression with drug sensitivity. **A** The heatmap shows the difference of the seven candidate FRGs expression among seven human kidney cancer cell lines. **B** The sensitivity of seven candidate FRGs in various drugs. The red dots represent sensitivity to the drug, while blue dots represent the opposite

We next utilized the CellMiner database to explore the expression of seven candidate genes in diverse kidney cancer cell lines, including 786-O, A498, ACHN, CAKI-1, RXF 393, SN12C, UO-31, and TK-10. We observed that the expression levels of these genes showed great heterogeneity in different cell lines (Fig. 10A). In addition, we also investigated the drug sensitivity of the seven candidate genes for the ccRCC patients using the GSCALite database. Four of them (CD44, SLC7A11, AKR1C2, and HMOX1) were highly related to drug sensitivity to a number of chemotherapy drugs (Fig. 10B), which provided direct support for drug targeted therapy.

## Discussion

It has been previously reported that ccRCC is a malignant disease; the pathogenesis may be related to the reprogramming of energetic metabolisms, such as tricarboxylic acid cycle, aerobic glycolysis, amino acids, fatty acids, and dysfunctional oxidative phosphorylation [34, 35]. Ferroptosis is a programmed cell death caused by iron-dependent lipid peroxidation and is different from other types of cell death [13, 36]. A wide variety of human diseases, including cancer, have been associated with the abnormal function of ferroptosis, and inhibition or upregulation of ferroptosis modulates the metabolic reprogramming of cancer cells [13, 37]. At present, few studies have been performed on ferroptosis in ccRCC, and the results also remain controversial [38]. Therefore, investigating the expression patterns of FRGs is critical to understand the role of ferroptosis in ccRCC patients.

In this study, we systematically explored the RNA-seq-expression and clinical information of ccRCC from the TCGA database. We found that 19 out of 61 FRGs were differentially expressed. To gain more insights into the functional roles of the differentially expressed FRGs in ccRCC, we carried out the functional enrichment analysis to investigate the associated biological processes and pathways. Functional annotation showed that many biological processes and pathways related to metabolism were enriched, especially lipid metabolism. The current consensus is that the execution of ferroptosis could result from the direct effects of lipid peroxidation [13]. Moreover, the increased lipid peroxidation is a principal mechanistic pathway in renal carcinogenesis induced by different chemicals. Therefore, we have reason to believe that ferroptosis may be involved in tumor metabolic reprogramming. We then categorized the ccRCC cohort into two clusters through consensus clustering analysis. Interestingly, the OS was dramatically different between the two clusters, suggesting that the levels of FRGs were significantly related to the prognosis of ccRCC patients.

Using Cox and Lasso regression analyses, we constructed a risk signature based on seven prognostic FRGs (CBS, HMOX1, CD44, AKR1C2, CHAC1, HMGCR, and SLC7A11). Every patient with ccRCC was assigned into high- and low-risk groups according to the median risk score. We noticed that the OS was shorter for the high-risk patients compared to the low-risk patients. The ROC curves revealed that the signature performed well. Recently, more and more studies have reported that the abnormal expression of the FRGs is involved in human cancer [15, 16, 37]. We further demonstrated that the signature-based risk score was highly associated with the ccRCC progression. It was also observed that the risk scores were higher in individuals with more advanced stage disease. Further stratified survival analysis showed that the high-risk group suffered a significantly lower OS rate than those in the low-risk group for all hierarchical cohorts. In addition, the signature of the seven FRGs was independent of other clinical factors. We then developed a nomogram that reduced the prognostic signature combined with other clinical parameters into a single numerical estimate of the probability of an event to predict the prognosis of every individual patient. Taken together, the above results showed the potential role of ferroptosis in ccRCC.

Our results showed that the expression level of CBS, CD44, AKR1C2, CHAC1, and SLC7A11was positively associated with the progression of ccRCC, while contrary results appeared in HMOX1 and HMGCR. Cystathionine *β*-synthase (CBS), a fundamental enzyme in l-cystathionine synthesis, catalyzes the condensation of serine and homocysteine to form cystathionine [39]. An increasing body of evidence points to the key roles of CBS in tumor progressions, such as ovarian cancer [40] and colon cancer [41]. However, another research regarded CBS as a negative regulatory role in hepatocellular carcinoma [42]. CD44 is an important cancer stem cell marker in tumors and implicates in malignant processes including cell motility, tumor growth, and angiogenesis [43]. In fact, CD44 has been observed in many human tumors and is associated with a poor survival rate [44]. Aldo-Keto reductase 1C2 (AKR1C2), a member of Aldo-Keto reductase subfamily, could mediate similar prostaglandin D2 conversion toward the accumulation of proliferative signals through PI3K/Akt signaling pathway to promote prostate cell proliferation [45]. In addition, Zhang et al. demonstrated that AKR1C2 could act as a targetable oncogene in esophageal squamous cell carcinoma via activating PI3K/AKT signaling pathway. ChaC glutathione-specific γ-glutamyl cyclotransferase 1 (CHAC1) is a proapoptotic γ-glutamyl cyclotransferase that depletes glutathione. There are few studies on CHAC1 at present, and its clinical significances and biological functions in tumors remain unknown. Solute carrier family 7 member 11 (SLC7A11; also known as xCT) is a cystine/glutamate antiporter that imports cystine into the cells while exporting glutamate [46]. SLC7A11 is highly expressed in human tumors, and its overexpression inhibits ROS-induced ferroptosis and abrogates p53(3KR)-mediated tumor growth suppression in xenograft models [47]. Results from a recent study suggested that smoking could induce the expression of SLC7A11, and the overexpression of SLC7A11 could promote lung tumor progression [48]. These results suggest that the four genes (CBS, CD44, AKR1C2, and SLC7A11) are significantly linked to tumorigenesis and progression, which are partly consistent with our research.

Heme oxygenase-1 (HMOX-1), a phase II enzyme that responds to electrophilic stimuli, has been reported to play protective or detrimental effects in different diseases, including cancers. HMOX-1 is elevated in a variety of human malignancies, indicating that it contributes to settle the tumor microenvironment for cancer cell growth, angiogenesis, and metastasis [49]. However, emerging evidence has revealed HMOX-1 functions as a negative regulator in erastin- and sorafenib-induced hepatocellular carcinoma and knockdown of HMOX-1 by specific shRNA increased erastin- and sorafenib-induced growth inhibition [23]. Hydroxymethylglutaryl-coenzyme A reductase (HMGCR), the rate-limiting enzyme in the mevalonate pathway, is generally believed to be a candidate metabolic oncogene [50]. For example, Li et al. found that HMGCR is upregulated in gastric cancer and promotes the growth and migration of the cancer cells by activating Hedgehog/Gli1 signaling [51]. Interestingly, another study reported that oral atorvastatin and its metabolites are detectable in human breast samples, suggesting that HMGCR may be directly inhibited in breast tumors [52]. It is evident from the above study that HMOX-1 and HMGCR have both tumor-promoting and tumor-suppressive properties. Clearly, further research will be needed to elucidate the role of cancers, especially ccRCC.

However, despite these encouraging results, there existed some limitations. Firstly, since the retrospective nature of this study, a prospective research is required to assess the potential applicability of our conclusions. Moreover, it is necessary to perform additional experiments to confirm the specific mechanism of key genes for clinical applications. In addition, some prognostic genes may be excluded because merely a single phenotype was considered to construct a prognostic signature in our study.

## Conclusions

In conclusion, we provided insights into the roles of FRGs in ccRCC and constructed a promising risk prognostic signature that exhibited potential as a biomarker of OS in ccRCC patients. The underlying mechanisms whereby differentially expressed FRGs exert their biological roles in metabolism-associated biological processes. We then established a novel promising prognostic nomogram incorporating for providing individualized survival prediction. Therefore, our constructed ferroptosis-related signature is of great clinical importance and may help facilitate personalized medicine in the clinical setting.

## Supplementary Information

**Additional file 1: Figure S1**. Survival analyses of each gene from the constructed prognostic risk signature based on the optimal cut-off expression value in the TCGA cohort. (A) CBS, (B) HMOX1, (C) CD44, (D) AKR1C2, (E) CHAC1, (F) HMGCR, and (G) SLC7A11.

**Additional file 2: Figure S2**. Validation of the prognostic risk signature in the ICGC databased of KIRP patients. (A) Kaplan-Meier curve analysis of overall survival of KIRC patients in high- and low-risk groups. (B) ROC curve analysis. (C) Risk score distribution, survival status, and lncRNA expression patterns for KIRC patients in high- and low-risk groups by the prognostic signature.

**Additional file 3: Figure. S3**. The single nucleotide variations (SNVs) and copy number variations (CNVs) of the seven candidate ferroptosis-related genes (FRGs) in the TCGA-KIRC dataset. (A) The type of genetic alterations of KIRC patients. (B) The variant classification of KIRC patients. (C) The SNV class of KIRC patients. (D) The characteristic of the frequently mutated genes. (E) The CNV alteration frequency of FRG in KIRC patients.

**Additional file 4: Table S1**. The list of ferroptosis-related genes.

## Data Availability

The gene expression profiles and clinical information datasets downloaded from The Cancer Genome Atlas (TCGA, https://cancergenome.nih.gov/) and the International Cancer Genome Consortium (ICGC, https://dcc.icgc.org/) databases.

## Abbreviations

AJCC: The American Joint Committee on Cancer
AUC: Area under the curve
BP: Biological processes
CC: Cellular component
C-index: Concordance index
DCA: Decision curve analysis
FRGs: Ferroptosis-related genes
GO: Gene ontology
GSEA: Gene set enrichment analysis
HR: Hazard ratio
ICGC: International Cancer Genome Consortium
KEGG: Kyoto Encyclopedia of Genes and Genomes
ccRCC: Clear cell renal cell carcinoma
LASSO: Least absolute shrinkage and selection operator
MF: Molecular function
OS: Overall survival
PPI: Protein-protein interaction
RBPs: RNA-binding proteins
RCC: Renal cell carcinoma
ROC: Receiver operating characteristic
TCGA: The Cancer Genome Atlas
