# Supplementary figures and images for: Prediction of overall survival based upon a new ferroptosis-related gene signature in patients with clear cell renal cell carcinoma
Source: World J Surg Oncol. 2022 Apr 14;20:120. doi: 10.1186/s12957-022-02555-9 (PMC9008912; doi:10.1186/s12957-022-02555-9)

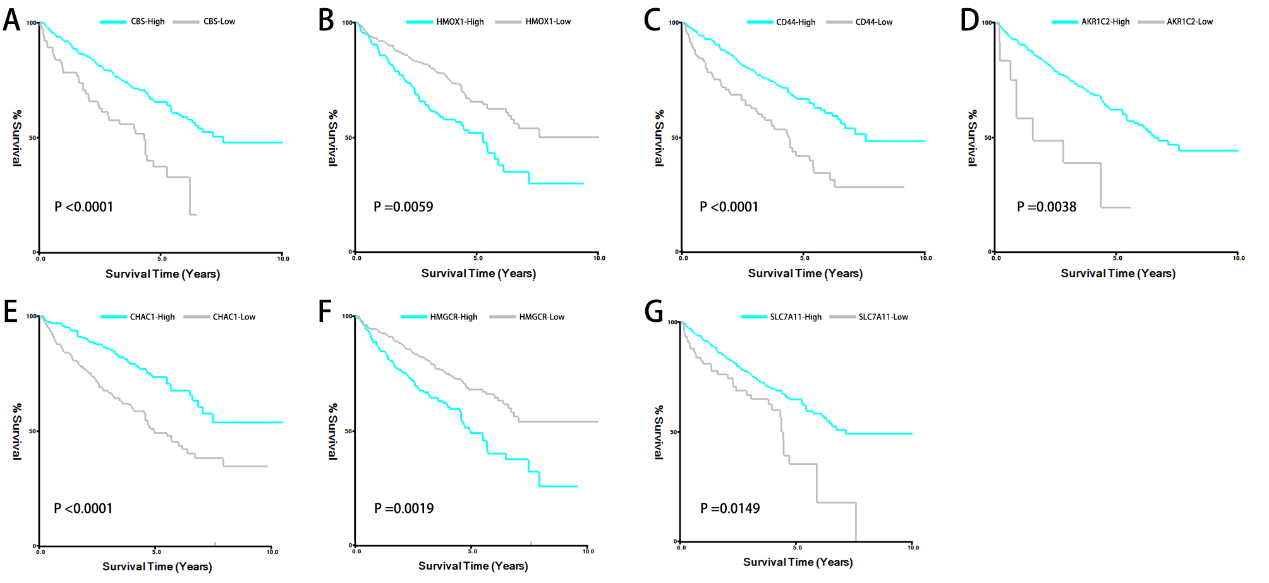

Supplement: Supplementary file 1 — Additional file 1: Figure S1. Survival analyses of each gene from the constructed prognostic risk signature based on the optimal cut-off expression value in the TCGA cohort. (A) CBS, (B) HMOX1, (C) CD44, (D) AKR1C2, (E) CHAC1, (F) HMGCR, and (G) SLC7A11. [file 12957_2022_2555_MOESM1_ESM.docx]

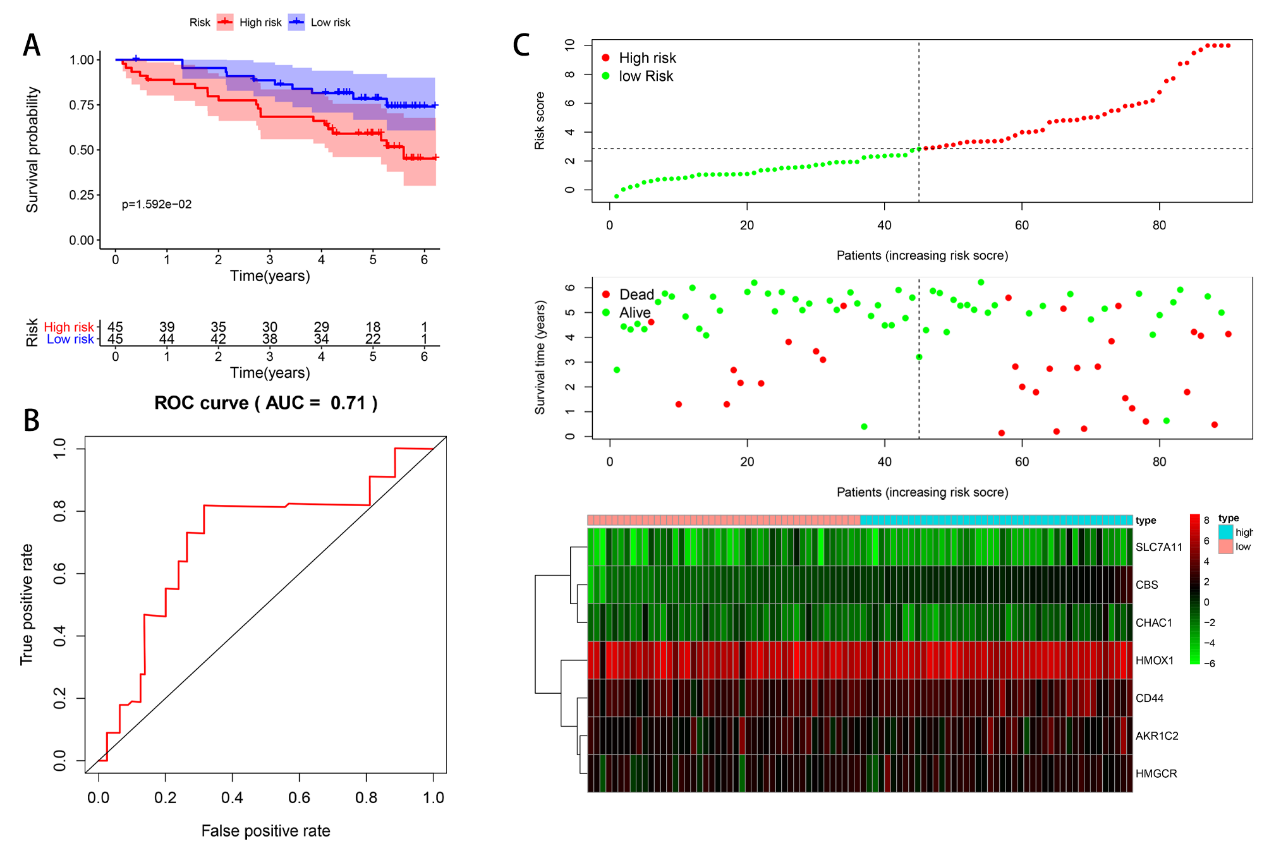

Supplement: Supplementary file 2 — Additional file 2: Figure S2. Validation of the prognostic risk signature in the ICGC databased of KIRP patients. (A) Kaplan-Meier curve analysis of overall survival of KIRC patients in high- and low-risk groups. (B) ROC curve analysis. (C) Risk score distribution, survival status, and lncRNA expression patterns for KIRC patients in high- and low-risk groups by the prognostic signature. [file 12957_2022_2555_MOESM2_ESM.docx]

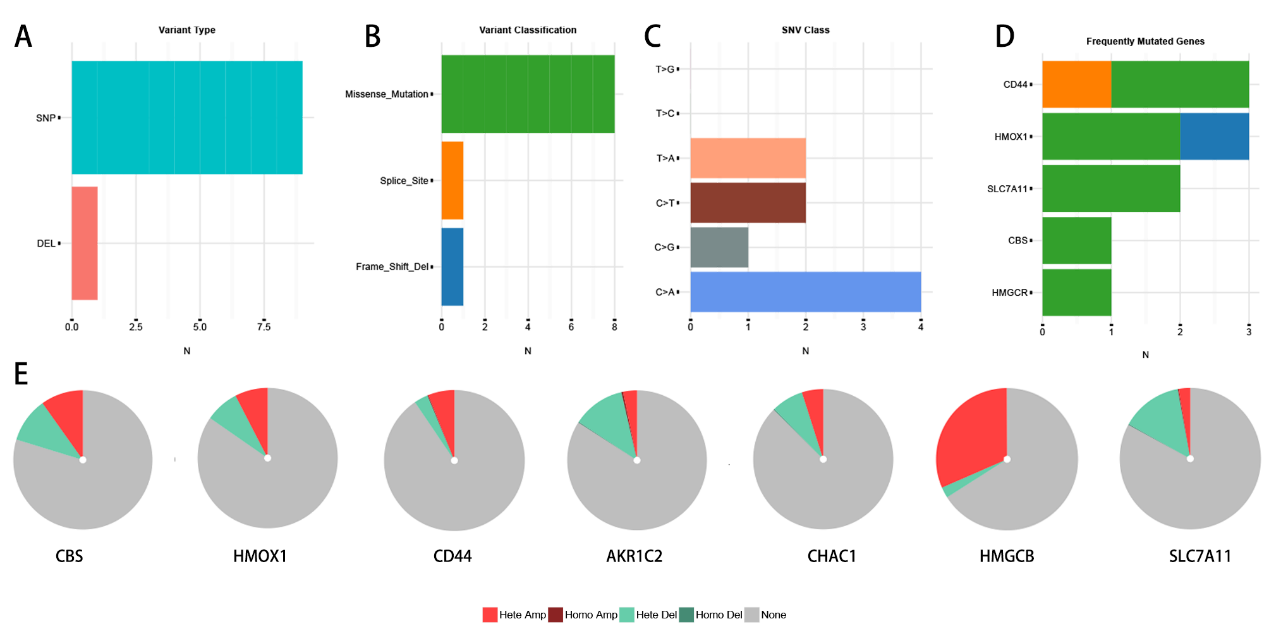

Supplement: Supplementary file 3 — Additional file 3: Figure. S3. The single nucleotide variations (SNVs) and copy number variations (CNVs) of the seven candidate ferroptosis-related genes (FRGs) in the TCGA-KIRC dataset. (A) The type of genetic alterations of KIRC patients. (B) The variant classification of KIRC patients. (C) The SNV class of KIRC patients. (D) The characteristic of the frequently mutated genes. (E) The CNV alteration frequency of FRG in KIRC patients. [file 12957_2022_2555_MOESM3_ESM.docx]
