# Supplementary material for: Prediction of overall survival based upon a new ferroptosis-related gene signature in patients with clear cell renal cell carcinoma
Source: World J Surg Oncol. 2022 Apr 14;20:120. doi: 10.1186/s12957-022-02555-9 (PMC9008912; doi:10.1186/s12957-022-02555-9)
Supplement: Supplementary file 4 — Additional file 4: Table S1. The list of ferroptosis-related genes. [file 12957_2022_2555_MOESM4_ESM.docx]

| **Ferroptosis-related genes** | **Name** |
| --- | --- |
| ABCC1 | ATP binding cassette subfamily C member 1 |
| ACACA | Acetyl-CoA carboxylase alpha |
| ACO1 | aconitase 1 |
| ACSF2 | acyl-CoA synthetase family member 2 |
| ACSL3 | acyl-CoA synthetase long-chain family member 3 |
| ACSL4 | acyl-CoA synthetase long-chain family member 4 |
| AIFM2 | apoptosis inducing factor mitochondria associated 2 |
| AKR1C1 | aldo-keto reductase family 1 member C1 |
| AKR1C2 | aldo-keto reductase family 1 member C2 |
| AKR1C3 | aldo-keto reductase family 1 member C3 |
| ALOX12 | [arachidonate 12-lipoxygenase](https://www.ncbi.nlm.nih.gov/gene/240) |
| ALOX15 | [arachidonate 15-lipoxygenase](https://www.ncbi.nlm.nih.gov/gene/246) |
| ALOX15B | arachidonate 15-lipoxygenase second type |
| ALOX5 | [arachidonate 5-lipoxygenase](https://www.ncbi.nlm.nih.gov/gene/240) |
| ATP5MC3 | ATP synthase membrane subunit c locus 3 |
| CARS | cysteinyl tRNA synthetase |
| CBS | cystathion ine beta synthase |
| CD44 | CD44 molecule |
| CHAC1 | ChaC glutathione- specific gamma-glutamyl cyclotransferase 1 |
| CISD1 | CDGSH iron sulfur domain 1 |
| CRYAB | heat shock protein beta 5 |
| CS | citrate synthase |
| DPP4 | dipeptidyl-dippeptidase-4 |
| EMC2 | ER membrane protein complex subunit 2 |
| FADS2 | fatty acid desaturase 2/acyl-CoA 6-desaturase |
| FANCD2 | Fanconi anemia comple mentation group D2 |
| FDFT1 | farnesyl-diphosphate farnesyltransferase 1 |
| FTH1 | ferritin heavy chain 1 |
| G6PD | glucose-6-phosphate dehydrogenas e |
| GCLC | glutamate-cysteine ligase catalytic subunit |
| GCLM | glutamate-cysteine ligase modifier subunit |
| GLS2 | glutaminase 2 |
| GOT1 | glutamic-oxa loacetic transaminase 1 |
| GPX4 | glutathio ne peroxidase 4 |
| GSS | glutathione synthetase |
| HMGCR | 3-hydroxy-3- methylglutaryl-CoA reductase |
| HMOX1 | heme oxygenase 1 |
| HSBP1 | heat-shock 27-k Da protein 1 |
| HSPB1 | heat shock protein beta 1 |
| IREB2 | iron response element-binding protein 2 |
| KEAP1 | kelch-like ECH- associated protein 1 |
| LPCAT3 | lysophosp hatidylcholine acyltransferase 3 |
| MT1G | metallothionein-1G |
| NCOA4 | nuclear receptor coactiva tor 4 |
| NFE2L2 | nuclear factor, erythroid 2 like 2 |
| NFS1 | cysteine desulfurase |
| NOX1 | NADPH oxidase 1 |
| NQO1 | quinone oxidoreductas e-1 |
| PEBP1 | phosphatidy lethanolamine-binding protein 1 |
| PGD | phosphoglycerate dehydrogenas e |
| PHKG2 | phospho rylase kinase ,g2 |
| PTGS2 | prostagla ndin-endoperoxide synthase 2 |
| RPL8 | ribosomal protein L8 |
| SAT1 | spermidine/spermine N1-acetyltra nsferase 1 |
| SLC1A5 | solute carrier family 1 member 5 |
| SLC7A11 | solute carrier family 7 member 11 |
| SQLE | squalene monooxygenase |
| STEAP3 | six-transm embrane epithelial antigen of prostate 3 |
| TFRC | transferrin receptor |
| TP53 | tumor protein 53 |
| ZEB1 | zinc finger E-box-binding homeobox 1 |
